# The Crosstalk between the Bone and the Immune System: Osteoimmunology

**DOI:** 10.1155/2013/617319

**Published:** 2013-09-26

**Authors:** Giacomina Brunetti, Giorgio Mori, Patrizia D'Amelio, Roberta Faccio

**Affiliations:** ^1^Department of Basic Medical Sciences, Neuroscience and Sense Organs, Section of Human Anatomy and Histology, University of Bari, 70124 Bari, Italy; ^2^Department of Clinical and Experimental Medicine, University of Foggia, 71100 Foggia, Italy; ^3^Department of Medical Sciences, University of Torino, 10126 Torino, Italy; ^4^Department of Orthopedics, Washington University School of Medicine, St. Louis, MO 63110, USA

Osteoimmunology is an interdisciplinary research field focused on the molecular understanding of the interplay between the skeletal and immune systems. Recent reports suggest that a large number of molecules affect both bone and immune cells. Furthermore, it has been demonstrated that immune cells express molecules modulating bone cell development and bone is known to provide important signal to the hematopoietic and immune cells, which develop in the bone marrow and then migrate to peripheral organs.

All these concepts are well represented in this special issue, starting from a large overview of bone cell differentiation, followed by the general mechanisms highlighting the interaction between bone and immune cells described by G. Mori et al. The effect of the numerous T-cell subsets on bone cells is very complex. In particular, a clear overview on Th17 and Treg and their involvement in bone diseases is presented by M. Wang et al., whereas a more detailed description of the negative regulatory feedback loop between osteoclasts and CD8+ T cells that contributes to homeostasis of both the skeletal and immune systems is provided by Z. S. Buchwald and R. Aurora.

The first identified pioneer cytokine regulating both bone and immune systems is RANKL, revised by N. Lo Iacono et al. The authors also report that a genetic defect impairing RANKL function leads to a peculiar form of an invalidating rare bone disease, named autosomal recessive osteopetrosis. In addition to RANKL, TNF-alpha is another cytokine known to play a fundamental role during osteoclastogenesis in the context of inflammatory arthritis, as described by H. Kitaura et al. Comprehensive reports detailing the interaction between bone and immune cells in the periodontal diseases and postmenopausal osteoporosis are reported, respectively, by A. Di Benedetto et al. and M. F. Faienza et al. The involvement of T cells in bone metastatic solid tumors has been carefully reviewed by I. Roato. Immune cell contribution in multiple myeloma bone disease is well described by A. Oranger et al. Understanding how bone and immune cells mutually interact with each other can give rise to new therapeutical targets as described by A. Corrado et al. in the context of rheumatoid arthritis. Moreover, T. Fujimura et al. demonstrated that, in invasive extramammary Paget's disease, the administration of bisphosphonates, privileged antiresorptive drugs, has also an immunosuppressive role in addition to blocking osteoclast activity.

Recent findings suggested that immune cells are critical mediators of the effect of different hormones on bone cells. In particular, G. Colaianni et al. analyzed the Pituitary/Immune/Bone Axis, highlighting the effect immune cell-mediated of hormones such as FSH and TSH on bone remodeling. Finally, further sustaining the role of bone cells on the regulation of haematopoiesis, C. L. Sesler and M. Zayzafoon demonstrated that the inhibition of NFAT activation in osteoblasts increases bone formation while decreasing B-cell development in the bone marrow microenvironment.

These manuscripts represent an exciting and insightful snapshot of current research on osteoimmunology. State-of-the-art and emerging future topics are highlighted in this special issue, which may help to advance the present research on the crosstalk between bone and immune cells.

*Giacomina  Brunetti* *Giorgio  Mori* *Patrizia  D'Amelio* *Roberta  Faccio*

